# Exploring the change management framework: An in-depth investigation

**DOI:** 10.1016/j.mex.2024.102978

**Published:** 2024-09-28

**Authors:** Sobia Asher, Muhammad Nafees, Tamkeen Syeda

**Affiliations:** a Computer Science & IT, Sir Syed University of Engineering and Technology, Karachi, Sindh, Pakistan; b Computer Science & IT, Karachi Institute of Economics and Technology, Karachi Sindh, Pakistan

**Keywords:** Requirement change, Project management, Requirement engineering (RE), Global software development (GSD) framework, Non-GSD Framework, Research Questions and Survey Research

## Abstract

Change management is part of Software Development Life Cycle (SDLC), particularly during the challenging stage of Requirement Engineering (RE). Managing requirements and incorporating changes can be complex and demanding, Having high impacts on project duration, cost, and ultimately project success and failure, cost, and ultimately, the success or failure of the software project. This research paper aims to explore and analyses various change management frameworks that contribute to the effectiveness of project completion, with a focus on minimizing time and cost estimates while maximizing product quality. Additionally, this study investigates the success and failure rates of software projects, specifically examining the identification of change management frameworks and addressing critical challenges within the context of Global Software Development (GSD). This review provides valuable insights and recommendations for implementing effective change management practices in software development projects that may facilitate future advancement in change management. This paper reviews various research papers and identifies major problems associated with each existing framework on Time constraints, Quick cost estimates, Verification of used and excluded needs, and customer information. We believe that the finding of this paper helps to address those challenges and reduce overall development time and increase the success rate of projects through the review of Requirement change management identification of a framework.

## Review Highlights

• This study investigates the success and failure rates of software projects, specifically examining the identification of change management frameworks and addressing critical challenges within the context of Global Software Development (GSD).
• This research paper aims to explore and analyze various change management frameworks that contribute to the effectiveness of project completion, with a focus on minimizing time and cost estimates while maximizing product quality.
• This paper reviews various research papers and identifies major problems associated with each existing framework on Time constraints, Quick cost estimates, Verification of used and excluded needs, and customer information.

## Graphical abstract

**Figure:**
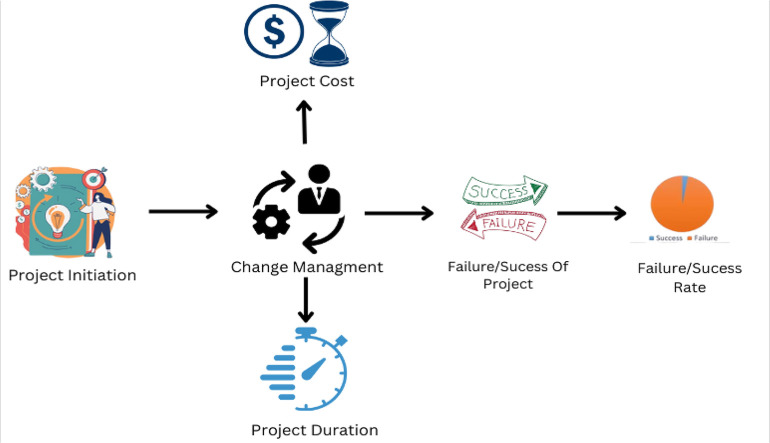

Specifications table

**Table:** 

Subject area:	Computer Science
More specific subject area:	Change Management Framework
Name of the reviewed methodology:	Research Questions and Survey Research
Keywords:	*Requirement Change; Project management; Requirement Engineering (RE); GSD (Global Software Development) Framework; Non-GSD Framework;*
Resource availability:	*Not applicable*
Review question:	***Research Q 1:****What are the key components of an effective Change Management Framework in the software development life cycle?* ***Research Q 2****: How does a well-defined Change Management Framework influence the success rate of software development projects?* ***Research Q 3:****What are the challenges and barriers in implementing a Change Management Framework in software development?* ***Research Q 4****: How can geographically distributed software development benefit from a Change Management Framework, and what unique challenges does it pose?* ***Research Q 5****: What best practices and case studies demonstrate the successful application of a Change Management Framework in the software development life cycle?* ***Survey Q 1:****How would you rate your understanding of the Change Management Framework?* ***Survey Q 2:****How effectively was the Change Management Framework explained to you?* ***Survey Q 3:****Did the materials used for exploring the Change Management Framework meet your expectations?* ***Survey Q 4:****How well did the training on the Change Management Framework align with your learning preferences?* ***Survey Q 5:****How confident do you feel about your ability to apply the Change Management Framework to real-world situations?* ***Survey Q 6:****How would you rate the relevance of the content covered while exploring the Change Management Framework?* ***Survey Q 7:****Were the practical examples provided during the training useful in understanding the Change Management Framework?* ***Survey Q 8:****How well do you think the Change Management Framework can be integrated into your current work processes?* ***Survey Q 9:****Did you find the exploration of the Change Management Framework engaging and interesting?* ***Survey Q 10:****How satisfied are you with the overall quality of the training on the Change Management Framework?* ***Survey Q 11:****How would you rate the accessibility of resources and support for your exploration of the Change Management Framework?* ***Survey Q 12:****To what extent did your interaction with peers and instructors enhance your understanding of the Change Management Framework?* ***Survey Q 13:****How well do you believe the Change Management Framework can address organizational change challenges in your workplace?* ***Survey Q 14:****How likely are you to recommend this exploration of the Change Management Framework to others?* ***Survey Q 15:****What suggestions do you have for improving the experience of exploring the Change Management Framework?*

## Background

In a purpose-driven organization, mission control in software engineering entails understanding how related professions like computer engineering interact. However, it might cause performance issues, especially when it comes to budget management. For software engineering projects to be successful overcoming these challenges is essential*;* it emphasizes the significance of evaluation as a tool for optimizing project development. Effective project control requires advanced planning, programming, and observation skills, along with resource management to overcome obstacles and achieve objectives. [1]*.*

This study discusses the importance of change control and project monitoring and evaluation in ensuring performance objectives are met and addressing operational issues Project control is essential for fostering team alignment, effective communication, and empowerment to manage project scope, time, and quality. It relies on strong collaboration skills and involves decision-making, monitoring, and adhering to customer expectations. Project control involves choosing the right project path, prioritizing customer satisfaction, and learning from past successes and failures. In order to successfully complete the mission by lowering the time and cost estimates while increasing effectiveness, this study investigates an alternative method to project management. The study highlights the benefits of leveraging previous organizational knowledge in improving project outcomes and aligning with business objectives.

This article explores the challenges of requirement change management in Global Software Development (GSD), where organizations aim to produce cost-effective products by leveraging distributed teams. GSD frameworks, such as central project management and local coordination, are discussed and compare GSD to the conventional project framework, emphasizing the value of precise planning and the viability of the Waterfall method for big teams with clear objectives.

## Method details

### Literature review

**Fig. 1:**
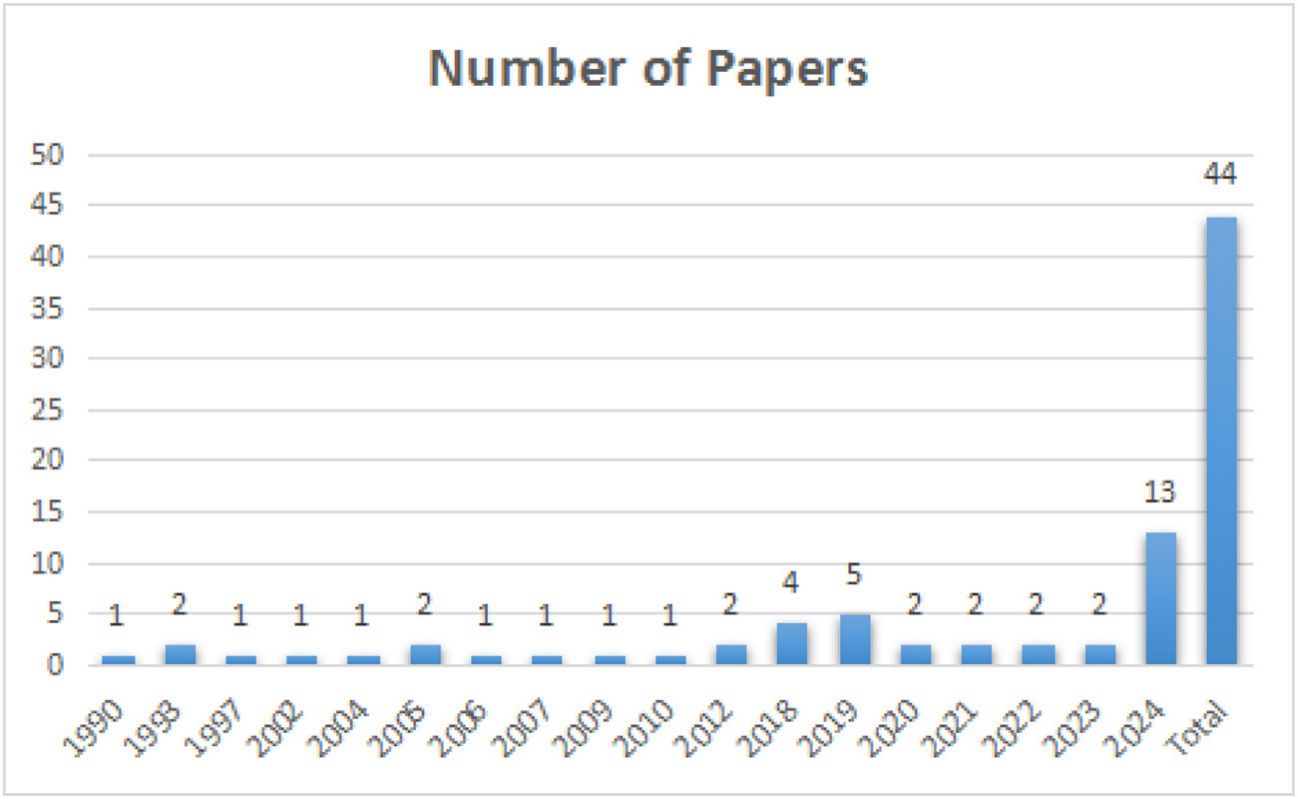

This literature review aims to identify different methods used in framework organizations to address challenges and factors during requirement change (RC). Hammer, Huffman, Rosenberg, Wilson, and Hyatt proposed a categorization of requirements into four levels based on their granularity. “Level 1” requirements are system-level requirements, while “Level 2” requirements are component-level requirements derived from “Level 1” requirements. “Level 3” requirements are sub-component level, and “Level 4” requirements are design-level with maximum changes [2]. Proposed a framework for categorizing, tracking, and notifying stakeholders about requirement status. The study also focuses on identifying tools and frameworks that support Global Software Development (GSD) project managers. A review of 15 relevant articles provided insights into change management frameworks, objective identification, and challenges in the research area. The figure below (Fig. 1) illustrates the publication years of the reviewed papers.

This studies awareness classify identifies requirement alternate management that is already described earlier than the beginning of the literature review.

**Table:** 

No	Author	Research Title	Proposed Research	Discussion	
				Conclusion	Future work
**LR-1**	William J. Kettinger, James T.C. Teng, Subhashish Guha	Business Process Change: A Study of Methodologies, Techniques, and Tools*	The purpose of this research is to conduct an inquiry into Business Process Reengineering (BPR). Methods, Techniques, and Tools (MTTs).	It helps to improve BPR practices.	Further research is based on Improvement needed by BPR professional business process and IS.
**LR-2**	Nafisa Osman, Abd-El-Kader Sahraou0i	A Software Requirement Engineering Framework to Enhance Critical Success Factors for ERP Implementation	This research purpose is to criticize the failure and success rate of a software project. This is focused on CS.	The conclusion of this paper is filling the study gap between CSF of ERP from the RE process.	Further research work is the enhancement of more achievable frameworks of RE and ERP, CSF
**LR-3**	Sharon McGee1 and Des Greer	A Software Requirements Change Source Taxonomy	The purpose of this paper defines the unpredictable changes during the software development cycle.	The outcome of this paper is to change Tigger taxonomy.	The future paining is investigating what kinds of requirements are more susceptible to change.
**LR_4**	Bano, Muneera Imtiaz, Salma Ikram, Naveed Niazi, Mahmood Usman, Muhammad	Causes of requirement change a systematic literature review	The purpose of this paper is to point out various reasons and frequency in the development phase of software.	The conclusion of this paper is to help save cost, reduce overall development time and increase the success rate of projects.	More experimental research is needed to identify and fully understand the causes of the change of need Keywords.
**LR-5**	S D P Harker and K D Eason	The Change and Evolution of Requirements as a Challenge to the Practice of Software Engineering	The purpose of this paper is to effective communication between users and designers.	The nature and causes of the change will determine the approach taken in a particular circumstance.	Another feature of these approaches is needed More experimental research for changing requirements
**LR-6**	Antje von Knethen	Change-Oriented Requirements Traceability. Support for Evolution of Embedded Systems	The purpose of this paper was to develop an accurate impact analysis that improves planning as well as implementing changes.	Three experimental studies show the real benefits of our approach An accurate set of feedback from the project planner can be used to accurately estimate the cost of implementing the desired change	According to experimental research, perspectives help impact analyses be more effective and efficient and assist more consistent change implementation. Explains our experimental findings and upcoming study.
**LR-7**	T. Hillman Willis and C. Richard Huston	Vendor Requirements and Evaluation in a Just-In-Time Environment	The purpose of this paper is to describe the attributes that a supplier should own to fulfil the role of JIT supplies.	The dimensional analysis model is particularly suitable for evaluating suppliers competing to furnish parts/materials in a just-in-environment environment.	In the future experimental research work needs to more accurately analyses models.
**LR-8**	AbhijitChakraborty, 2 MrinalKanti Baowaly, 3 Ashraful Arefin, 4 Ali Newaz Baha	The Role of Requirement Engineering in software development cycle	This paper shows an effective engineering method of need. This paper examines the hospital to be a case study for which a software system has been developed keeping in view the approach mentioned.	The OSDM widely used approach takes an object-based approach.	According to this viewpoint, a domain-based method is suitable for capturing users' needs and wants as well as its boundary, which minimizes error on subsequent phases and maintenance costs.
**LR- 09**	Bhatti, M. W., Hayat, F., Ehsan, N., Ishaque, A., Ahmed, S., &Sarwar, S. Z.	An Investigation of Changing Requirements concerning Development Phases of a Software Project	It highlights the significant correlation between design and testing phase changes, emphasizing the need for effective change management to ensure successful software development.	The changing requirements of the software project are analyzed in detail through narrative data, spearman's RHOs, and regression analysis.	In the future experimental research work needs more accurate static analysis.
**LR-10**	Khan, Arif Ali Basri, Shuib Dominic, P. D.D.	A proposed framework for requirement change management in global software development	The aim of this study is to propose a framework for RCM in geographically distributed software development systems and describe the identification of the communication risks, their causes, and effects during RCM in GSD	The proposed RCM framework is aimed at improving understanding of the roles, artifacts, and activities involved in GSD, especially from the perspective of the management system of change	In addition, some routes will be proposed to reduce communication risks which have been highlighted during RCM in GSD projects
**LR- 11**	Zainab ShehzadI,Farooque Azam, M. Waseem Anwar, Iqra Qasim	A Novel Framework for Change Requirement Management (CRM) In Agile Software Development (ASD)	This paper proposes how to categorize requirements, track implemented/unimplemented requirements, Save/Update requirements in the repository.	The proposed CRM framework applies to all kinds of software organizations, especially for the organization dealing with agile procedures.	In the future, we intend to extend the proposed framework to include the risk identification phase associated with the CRM process.
**LR- 12**	Akbar, Muhammad Azeem	SRCMIMM Managing Requirements Change Activities in Global Software Development.	The objective of this research work is to develop a model for the successful implementation of RCM activities in the GSD environment.	This study identifies the successful factor and challenges through the SLR approach concerning the “project administration” category	In future, we will conduct real-world study.
**LR-13**	Agyeman Addai, Daniel	A Cloud-Based Framework For Managing Requirements Change In Global Software Development	In the model, all stakeholders have a single platform from which they can communicate, collaborate, and control the processes involved in managing change.	The purpose for the investigation into the difficulties of managing requirements change in global software development and the crucial role of collaboration technologies, together with its significant conceptual contributions, has been reiterated.	The role of proxy client and its implications on global software development activities has been a beneficial area for future study.
**LR-14**	M Azeemakbar,Mshafiq,drnasraullah	AZ-Model of software requirements change management in global software development	This study proposed a comprehensive framework for the successful implementation of RCM activities in Global Software Development (GSD) namely “AZ-Model of RCM”.	The proposed model AZ- Model of RCM has the capabilities to handle the RCs efficiently through specialized project management and strong time boxing.	In the future, it is planned to conduct a systematic literature review (SLR) to identify the barriers to the RCM process in the context of GSD.
**LR-15**	M Azeem Akbar, Nasrullah, Shameem, Amna Maqboo, Khizer Abbas	Investigation of Project Administration related challenging factors of Requirements Change Management in global software development: A systematic literature review	In GSD the software development activities are carried out across geographical boundaries. The objectives of GSD is to minimize the development cost and compete with the market by using the globally available resources	Finally, it results that all sized organizations experienced a few different challenges while implementing the RCM process in the GSD environment.	The primary objective of this study is to develop software requirements change management and implementation maturity model (SRCMIMM).

This article identifies and validates 25 major problems in Requirements Change Management (RCM) for Global Software Development (GSD) projects by a thorough literature analysis and questionnaire survey. Organizational size (small, medium, large) as well as the organizations of the clients and vendors were used to categories these difficulties. The study intends to assist GSD organizations in efficiently addressing RCM concerns and discovered a moderate correlation between the survey rankings of these obstacles and the literature [3], the iSPIN framework, which greatly increases productivity in tasks like requirements analysis and RFP answers, is presented in this paper as a solution to quality difficulties in early SDLC phases. This is achieved by utilizing AI for knowledge extraction and intelligent recommendation. This study suggests an all-encompassing method for replacing laborious, document-centric processes with automated, machine-first solutions [4]. This paper presents a thorough analysis of several SDLC approaches, such as Waterfall, V-Model, Iterative, Agile, and Hybrid, and addresses their features, advantages, disadvantages, and common uses. Additionally, it examines the use of decision support matrices to choose the best technique for particular projects and contrasts traditional SDLC with Agile methodologies [5]. A digital performance-based appraisal system (d-PBAS) for professors at JSS Academy of Higher Education & Research, Mysuru, was designed, developed, and validated using UGC criteria. The paper reports on this process, demonstrating excellent user satisfaction and efficiency. This novel approach streamlines the yearly assessment of academic performance, improving precision and cutting down on processing time [6]. This study uses the NIST Cyber security SDLC Framework to analyses and describe the integration of security across multiple development models, looking at how different Software Development Life Cycles (SDLCs) incorporate security measures. In light of the always changing threats and vulnerabilities, it emphasizes how crucial it is to incorporate information security throughout the software development process [7]. Through a thorough literature research and case studies, the study creates a Requirements Change Management and Implementation Maturity Model (SRCMIMM) for the Global Software Development (GSD) industry, addressing crucial success factors and difficulties to improve RCM effectiveness and efficiency. The model is intended to support practitioners in evaluating and overseeing RCM endeavors at varying stages of development [8]. The programme "Routonomics," created by Aviation Management Consultants to help airlines create long-term business plans, is used as a case study in this study to investigate how the Waterfall methodology is applied in the software development life cycle (SDLC). In order to demonstrate the usefulness of the SDLC, the research also included a simulated development process [9]. The emphasis of this article is on confidentiality, integrity, and system availability as we look at how the SDL-Agile Method is being used in e-commerce development to improve security across the Software Development Life Cycle [10]. Using Design Science Research (DSR) approaches, this study offers an Integrated Digital Transformation System Framework (IDTSF) to solve issues in the initiation, execution, and governance of digital transformation. The goal of IDTSF is to improve and streamline business operations. In order to effectively address user needs and steer clear of typical digital transformation issues, the IDTSF seeks to connect formal processes such as Design Thinking, Agile SDLC, and Waterfall SDLC [11]. An analysis of rigorous and agile software development life cycles (SDLCs) for business process management systems (BPMS) is presented in this study, along with a critique of existing approaches and recommendations for improvement and standardization. Practitioners and academics of BPMS report satisfactory utility; ease of use, and value, according to an empirical pilot evaluation [12]. In order to promote the use of Large Language Models (LLMs) in RE tasks, this study examines these developments and gives experimental findings. With advanced techniques like BERT proving useful in classifying functional and non-functional requirements, artificial intelligence, in particular Natural Language Processing (NLP), greatly improves Requirement Engineering in the Software Development Life Cycle (SDLC) by addressing traditional inefficiencies [13], In order to improve requirements change management (RCM) procedures for global software development (GSD), this study examines human-related success factors (HSFs) and difficulties (HCHs) in the process. It does this by identifying key elements and putting forth a theoretical framework. The study finds ten HSFs and ten HCHs through a systematic literature review (SLR), of which five HSFs and four HCHs are considered essential for RCM implementation in GSD contexts [14].

Requirement change management is an important aspect of the requirement engineering phase and plays a crucial role towards project success. Changed customer needs, market trends, and organizational or business needs are the main reasons of RCM [15]. The adoption of a new SDLC model often necessitates a cultural shift within organizations. It is essential to foster a culture of collaboration, innovation, and continuous improvement to support the transition [16]. Change management strategies should be employed to communicate the rationale behind the new model, address concerns, and garner buy-in from stakeholders at all levels of the organization [17]. Equipping team members with the necessary skills and competencies to effectively operate within the new SDLC model is critical. Training programs should be designed to familiarize personnel with computational intelligence techniques, agile methodologies, and other relevant tools and practices [18]. The successful implementation of the new SDLC model relies on robust infrastructure and appropriate tooling to support development activities. Organizations need to invest in modern development environments, collaboration platforms, version control systems, and integrated development tools that facilitate agile practices, automation, and computational intelligence integration [19]. Evaluation and validation are integral components of the proposed Software Development Lifecycle (SDLC) model, ensuring its effectiveness, efficiency, and alignment with project objectives [20]. Low-quality change management is the major barrier in the accomplishment of software systems and the most likely reason for system failure. Therefore, it is necessary to acquire earlier and more basic knowledge to accomplish better change management [21]. Resistance management is a critical factor for RCM. The internal politics of the organizations badly effect in the process of RCM, as some of personnel do not want changes in requirements due to their personal benefits. Though, resistance management is important to elicit the pure requirement and to perform the activities of change management in an effective way [22]. Implementation of equally distributed security measures in every phases of software development phases are crucial and has significantly reduce vulnerability of the system as well as reducing the cost and time consume to develop the system [23]. The change management process has the following four phases:

➢ Initial issue evaluation
➢ Preliminary analysis
➢ Detailed change analysis
➢ Implementation

In the first step, the comments gathered from the stakeholders are validated and entered into a database as change requests. If a change request addresses a problem that is within the scope of the technical baseline, and has not been addressed before, a change proposal will be generated. In the second step, an analysis plan is formulated which describes the problem of the change proposal in detail. If this plan is approved by a change control board, then many potential solutions will be developed, from which one will be selected for implementation. This solution then needs to undergo further approval. In the third step, the solution approved by the preliminary analysis report is further analyzed against the technical baseline to determine the impact on the system in detail and the changes required. In the last step, the technical baseline is modified according to the change proposal and the change request is closed [24]. When creating a change management plan, understanding the social setting (e.g., employees’ experiences and perceptions of culture) in the R&D department is essential [25]. The ML model refinement and feature engineering are integrated into the Software Development Life Cycle (SDLC) process encompassing the various stages involved in the design and development of the software [26]. The SDLC process varies for different projects and teams, the fundamental phases remain the same [27].

## Methodology

The Software Development Life Cycle (SDLC) requires the use of change management frameworks in order to successfully manage changes, reduce interruption, and guarantee project deliverables. These well-known change management frameworks have been modified for the SDLC using straightforward.

1. ADKAR Model (Awareness, Desire, Knowledge, Ability, and Reinforcement):
  Definition: Assists team members in adjusting to changes in the Software Development Life Cycle (SDLC) by focusing on controlling individual change via designated stages.
2. Example in SDLC:
  A new coding standard must be embraced by the development team. Make developers aware of the reasons the new standard enhances the quality of the code. Desire: To encourage change, highlight advantages such a shorter debugging time. Knowledge: Conduct instruction on the most recent standards. Proficiency: Gain experience applying the standards in a regulated setting or on simple assignments. Reinforcement: To guarantee conformance, evaluate code frequently and provide comments.
3. Kotter's Eight-Step Change Model Definition: An organized method that emphasizes momentum and urgency for implementing change gradually.
4. Example in SDLC:
  Including DevOps techniques in the process of developing. Establish Urgency: Describe why enhanced cooperation and quicker deployment are necessary. Create a Potent Alliance: Obtain assistance from operational personnel and important developers. Develop a Change Vision: Describe how releases will be streamlined using DevOps. Share the Vision: To inform everyone, use documentation and meetings. Eliminate Barriers: Take care of issues like resource availability and tool compatibility. Obtain Quick Wins: Demonstrate early success with a more dependable, quick deployment. Expand on the Modification: Apply DevOps techniques to other projects. Integrate DevOps into regular procedures and guidelines to help anchor the cultural changes.
5. Lewin's Change Management Model Definition: Unfreeze, Change, and Refreeze is a straightforward three-step procedure.
6. Example in SDLC:
  Transferring an old system to a fresh platform.
  Unfreeze: Explain why updating the system is necessary to reduce security threats.
  Change: Get the team involved in starting the code and data migration to the new platform.
  Refreeze: Make sure the new platform is utilized consistently by training users and documenting the new system procedures.
7. McKinsey 7-S Framework Definition: To guarantee successful change, an organization's seven essential components must be aligned.
8. Example in SDLC:
  Presenting the Agile project management process.
  Strategy: Arrange to put Agile into practice to improve adaptability.
  Structure: Modify the roles within the team, e.g., assigning a Product Owner and Scrum Master.
  Systems: Introduce new agile task tracking tools, such as Jira.
  Shared Values: Encourage a collaborative and ever-improving culture.
  Proficiency: Educate groups on agile methodologies, such as daily stand-ups and sprint planning.
  Style: Motivate the leadership to take on a more assisting and facilitating role.
  Employees: Verify that the appropriate individuals are assigned to positions that align with agile methodologies.
9. Bridges' Transition Model Definition: Emphasizes the human aspect of change, offering guidance through the phases of transitioning, accepting change, and letting go.
10. Example in SDLC:
  Switching to automated testing from manual testing.
  Closing, Losing, Letting Go: Respond to testers who are concerned about their positions.
  The Neutral Zone: Conduct some manual testing while also offering instruction in automation.
  The Brand-New Start: Completely switch to automated testing and recognize accomplishments like higher test coverage and fewer defects.
  These frameworks ensure smoother transitions and minimize resistance by helping to handle SDLC changes efficiently.

### Case study

Here's a brief, small-scale example to help you understand how case studies can effectively validate theoretical claims and enhance credibility:

Framework: Agile Project Management Theoretical Claim: Agile improves project flexibility and customer satisfaction.

Case Study Example: A small software development company used Agile to manage a mobile app project; prior to adopting Agile, they used a traditional Waterfall approach, which resulted in delayed deliveries and unmet client expectations. By adopting Agile, they conducted bi-weekly sprints, engaged in continuous feedback with the client, and made iterative adjustments. The project was completed 20 % faster than previous projects, client feedback was incorporated in real-time, and customer satisfaction increased by 30 %. This case directly supports the claim that Agile enhances project flexibility and client satisfaction.

### Change management effectiveness to project success metrics

By connecting change management frameworks to important project success measures like cost, time, and quality, their efficacy is shown. By eliminating wasteful spending, these frameworks maximize economic effectiveness, enhance quality by guaranteeing consistent procedures and results, and improve time management. The direct effect of change management on the overall performance of a project becomes evident and quantifiable by measuring these particular criteria.

### Investigation for application for change management

Regression modelling and correlation analysis are two statistical techniques that are crucial for analyzing the connections between many elements that affect how effective change management is. By determining which variables are most important and how they interact, an understanding of these interactions can assist organizations in implementing change management techniques that are more successful.

**1. Analysis of Correlations**

A statistical technique for determining the direction and degree of a relationship between two or more variables is correlation analysis. Determining the extent to which different elements are related to the effectiveness of change initiatives is helpful in the context of change management.

### Important elements

The goal is to evaluate the degree of correlation between several factors, including training quality, leadership effectiveness, employee engagement, communication efficacy, and change management results.

### Correlation types

Measures the linear relationships between continuous data using Pearson correlation. When evaluating relationships between ranked variables, Spearman's Rank Correlation is helpful, especially in cases of non-linear associations. The ordinal relationship between two variables is measured using Kendall's Tau, which is robust against outliers.

### Uses of change management

Determining Important Factors Which elements—like leadership support or communication frequency—are most closely linked to successful change outcomes can be found using correlation analysis. Comprehending interdependencies facilitates the understanding of the relationships between different elements, such as the effect of a leader's style on the involvement of employees throughout transformation. Risk assessment: Organizations can foresee possible obstacles in the process of transformation by identifying characteristics that are weakly connected.

**Example:** The significance of effective training programs can be shown by examining the relationship between the caliber of staff training and the rate at which change is adopted.

**2. Regression Modelling**

A strong statistical method for examining the relationship between one or more independent variables (predictors) and one or more dependent variables (outcomes) is regression modelling. It surpasses correlation by quantifying each predictor's influence on the result in addition to assessing the direction and intensity of correlations.

### Important elements

Goal: To measure each factor's impact and forecast how change management projects will turn out based on a variety of criteria.

### Regression model types

Investigates linear correlations between variables; good for preliminary examinations. In order to determine how several independent factors together affect the dependent variable, multiple regression is used. When the outcome variable is categorical, such as the success or failure of change initiatives, logistic regression is used. With the help of hierarchical regression, one may evaluate the incremental effects of variables such as leadership or training on the success of transformation. Structural Equation Modelling (SEM): Frequently used to test theoretical models of change management, SEM combines factor analysis and regression to assess complicated interactions among variables.

### Change management applications

Measuring Impact The precise contribution of each component (such as leadership support and training efficacy) to the success of change management can be estimated using regression models. Predictive analysis involves making predictions about how change initiatives will probably turn out in various contexts, such as high vs low staff involvement.

Optimization is the process of determining which variables to increase or decrease in order to get better results.

**Example:** An organization may discover through multiple regression analysis that while good communication and leadership support are both important predictors of change success, the influence of leadership support is twice as great.

### Steps to apply these methods in change management

1. Describe the variables: Determine the important variables (independent variables) that affect change management, such as the success of the change initiative and the dependent variable, such as employee morale, training quality, and communication effectiveness.
2. Collect Data: Use organizational metrics, interviews, and surveys to compile both quantitative and qualitative data.
3. Pre-Processing: Make sure the data satisfies the presumptions of the statistical techniques being used, clean it up, and deal with missing numbers.
4. Analyze early links between components by conducting correlation analysis.
5. Regression Modelling: Create regression models to estimate each factor's influence and forecast results. Use statistical tests like as residual analysis, p-values, and R-squared to validate your models.
6. Interpret Results: Make strategic decisions in change management by using the results to comprehend the importance and magnitude of each element.
7. Execute Insights: Utilize the learnt lessons to maximize change management tactics by concentrating resources on the elements that will have the biggest effects.

### Advantages of applying statistical techniques

Evidence-Based Decision Making: Offers change management tactics a data-driven foundation. Improved Predictive Capability: Project change projects' likelihood of success in various scenarios. Resource Optimization: Increase the effectiveness of change management procedures by concentrating efforts on the most important variables. Continuous Improvement: Over time, regular analysis aids in the improvement of change management tactics as they adjust to fresh situations and revelations.

### Effective change management framework

1. Identify the Change Objective: Clearly state the purpose of the change. Align the aims of the organization with the transformation objectives. Analyze the change's impact and scope, taking into account the departments, procedures, and systems that will be impacted. To ascertain the effects on stakeholders, perform an impact analysis. Establish quantifiable success criteria in order to evaluate the change's efficacy.
2. Engagement and Analysis of Stakeholders Determine Stakeholders: Enumerate all people, groups, and outside entities that the change may affect. Stakeholder Mapping: Divide stakeholders into groups according to their degree of impact, influence, and interest. Stakeholder matrices are one technique that can be used to priorities engagement initiatives. Planning Communications: Create a focused communication strategy that highlights the advantages of the shift and responds to the concerns of each stakeholder group.
3. Assessment of Change Readiness Analyze the organization's existing situation to see if it is ready for change, taking into account technological, structural, and cultural aspects. Readiness surveys and interviews: To find out how employees feel about change and how ready they are for it, conduct focus groups, surveys, and interviews. Create a readiness scorecard to measure the degree of preparedness in each department inside the company.
4. Planning for Change and Developing Strategies Revise the Plan and Vision: Create a concise vision statement that describes the post-change future condition. Create a plan with deliverables and important benchmarks.

**Allocating Resources**: Determine which resources are required, such as staff, funds, and technology, then make sure they are distributed wisely.

**Risk management**: Make a plan for managing risks that includes information on possible hazards, their effects, and ways to mitigate them. Create a risk register and keep an eye on it during the change process.

5. Interaction and Instruction Communication Channels: To distribute information consistently and openly, use the right channels of communication (such as emails, meetings, the intranet, etc.).

**Instructional Plans**: Create training materials suited to various positions. Provide staff with the tools they need to handle the new procedures by providing them with job aids, e-learning, and practical training.

**Mechanisms of Feedback**: Establish feedback loops (question and answer sessions, suggestion boxes, surveys) to get continuing input and quickly address issues.

6. Execution and Observation create a thorough implementation strategy that includes deadlines, roles and duties, and key performance indicators (KPIs).

**Pilot Examining**: Before a full-scale launch, conduct pilot testing in a controlled environment to validate change initiatives and discover areas that require improvement.

**Performance Monitoring**: Monitor progress made in relation to the implementation strategy using dashboards and monitoring tools. Based on current data, make any necessary adjustments.

7. Strengthening and Maintaining Celebrate achievements: To keep the momentum continuing and inspire continued support, mark and commemorate significant achievements. Constant Enhancement: Create a continuous improvement process to assess the results of the changes on a regular basis and make incremental improvements. Integrating Modifications: To ensure that the change is maintained, create governance structures, rules, and procedures that support the new style of functioning.
8. Evaluation Following Change Post-Implementation Review: Evaluate the change process in detail, comparing it to the previously established success criteria. Learnings: Record the lessons learnt, emphasizing the things that went well and the things that should be done better for further change projects.

Reporting: Provide stakeholders thorough reports that provide a summary of the change's results, accomplishments, difficulties, and next steps.

### Key principles for practitioners

**Transparency**: To foster trust and lessen opposition, keep the lines of communication open.

**Adaptability**: Be ready to modify plans of action in the face of unforeseen difficulties.

**Empathy**: Acknowledge that change involves people. Respond to worries and anxieties with compassion. Make sure that the messaging is consistent at all organizational levels. With the help of this framework, practitioners may lead effective change projects while reducing disruptions and increasing engagement in an organized manner.

a. This study's online survey was carried out to ensure participant convenience and accessibility by reaching a broad and diversified population through the use of digital channels.
b. Shown in results.
c. Based on their relevance and subject-matter expertise, we chose poll respondents from academics, software houses, cyber security, and information technology. The field of information technology (IT) is at the forefront of technical breakthroughs and has a direct impact on the creation and application of digital solutions. IT participants offer insightful information about the most recent technologies, problems, and trends shaping the technology industry.

**Software homes**: An essential component of the software development lifecycle are software homes. Their involvement in the development, testing, and implementation of software solutions is essential, and their opinions are vital in comprehending industry norms, quality requirements, and user experience issues.

**Cyber security (Four Secure):** Participants from cyber security companies, like Four Secure, contribute specific knowledge on protecting software and IT systems, given the growing significance of data protection and digital security. Their knowledge aids in locating security holes and suggesting safe development techniques.

**Academics**: Contributions from academics add a theoretical viewpoint and insights from recent cutting-edge research to the conversation. They can bridge the knowledge gap between academia and business practice by examining novel ideas and offering a fair assessment of how developing technology should be used in real-world scenarios. These industries were selected to provide a thorough and well-informed research since each group offers distinctive, insightful viewpoints that advance our knowledge of the topic as a whole.

d. Because of the large number of replies we got, our study analysis will use a broad and representative dataset.
e. The findings of the poll should be received in two months. This process takes about sixty days to complete.

This review paper was conducted using the following methodology:

### Research questions

The survey was conducted using the following research questions:

**RQ 1:** What are the key components of an effective Change Management Framework in the software development life cycle?
**RQ2**: How does a well-defined Change Management Framework influence the success rate of software development projects?
**RQ3:** What are the challenges and barriers in implementing a Change Management Framework in software development?
**RQ4**: How can geographically distributed software development benefit from a Change Management Framework, and what unique challenges does it pose?
**RQ 5**: What best practices and case studies demonstrate the successful application of a Change Management Framework in the software development life cycle?

**Research Question 1**: What are the key components of an effective Change Management Framework in the software development life cycle?

Change identification, impact analysis, stakeholder communication, and process integration are all components of an effective Change Management Framework. These components work together to ensure smooth transitions and minimal disturbance throughout the software development life cycle.

**Research Question 2**: How does a well-defined Change Management Framework influence the success rate of software development projects?

A well-defined Change Management Framework improves the success percentage of software development projects dramatically. It guarantees that changes are appropriately analyzed, documented, and integrated, which reduces risks and improves project predictability.

**Research Question 3**: What are the challenges and barriers in implementing a Change Management Framework in software development?

Implementing a Change Management Framework presents challenges such as resistance to change, a lack of resources, and insufficient communication. Overcoming these roadblocks is critical for successful framework implementation.

**Research Question 4**: How can geographically distributed software development benefit from a Change Management Framework, and what unique challenges does it pose?

A Change Management Framework supports geographically distributed software development by providing efficient communication and collaboration among remote teams. However, difficulties such as time zone differences and cultural differences should be addressed by the framework.

**Research Question 5**: What best practices and case studies demonstrate the successful application of a Change Management Framework in the software development life cycle?

Comprehensive change documentation, proactive risk management, and clear communication routes are all best practices. Case studies show how organizations have used effective Change Management Frameworks to achieve successful software development outcomes.

A review for this paper was performed by collecting related articles from various online resources.

Analytical Hierarchy Way strategy: During the development phase of the project, the research uses the Analytical Hierarchy Way (AHP) strategy to define the scope of project outcomes and its impact on project goals. AHP aids in identifying, defining, and managing stakeholder requirements for the project's scope [28]. The study recognizes the difficulty of adjusting requirements during the software Development life cycle (SDLC), which has an impact on costs, budgets, and organizational goals.

Management of Change: The research focuses on change management, which is the act of addressing organizational changes and alterations in an efficient manner. Requirements changes come with implications for cost, time, and quality. As these alterations spread from one phase to another, their impact grows. The study's goal is to identify and resolve any risks that could arise from requirement changes, such as higher prices, scheduling problems, unstable demand, and lower quality [29].

GSD is a well-liked strategy in which businesses employ distributed development teams to create software that is both affordable and of high quality. The study investigates low-cost methods of development that make use of skilled labor. Benefits include a 24/7 development methodology that spans time zones and emphasizes flexibility and exploration to address pain areas, as well as shorter project marketing times [30].

Non-GSD Model: The non-GSD model is taken into account in circumstances when time estimation, budget computation, and scheduling are critical. This model may not need substantial architecture and art because it prioritizes costs. In order to provide a customized and effective development process, the study investigates the integration of both GSD and non-GSD models, depending on the project objectives.

Waterfall Method: Senior software engineers' preference for a "system-driven process" is explored in connection to the Waterfall method, which is recognized for its systematic and structured approach [31]. The study emphasizes the need of having a clear strategy and comprehending the tasks, their order, and the reasons for them. The Waterfall approach is seen to be appropriate for big organizations with well-defined goals and a thorough comprehension of the project scope.

## Survey research

Change management frameworks help firms navigate and accomplish transformative efforts by providing a disciplined strategy. These frameworks, which are based on well-established approaches such as Kotter's 8-Step Process and Lewin's Change Model, provide a road map for effective change projects. From strategy to implementation, their structured methods contribute to better change management strategies and improved organizational adaptability. Surveys are useful research tools because they provide a systematic way of acquiring data from varied populations. They allow researchers to efficiently collect quantitative and qualitative data, making them an essential component of empirical studies. Survey design and administration necessitate thorough thought in order to assure the validity and dependability of the data, which is critical for deriving meaningful findings in research.

**Table:** 

S No	Survey Questions
S-1	How would you rate your understanding of the Change Management Framework?
S-2	How effectively was the Change Management Framework explained to you?
S-3	Did the materials used for exploring the Change Management Framework meet your expectations?
S-4	How well did the training on the Change Management Framework align with your learning preferences?
S-5	How confident do you feel about your ability to apply the Change Management Framework to real-world situations?
S-6	How would you rate the relevance of the content covered while exploring the Change Management Framework?
S-7	Were the practical examples provided during the training useful in understanding the Change Management Framework?
S-8	How well do you think the Change Management Framework can be integrated into your current work processes?
S-9	Did you find the exploration of the Change Management Framework engaging and interesting?
S-10	How satisfied are you with the overall quality of the training on the Change Management Framework?
S-11	How would you rate the accessibility of resources and support for your exploration of the Change Management Framework?
S-12	To what extent did your interaction with peers and instructors enhance your understanding of the Change Management Framework?
S-13	How well do you believe the Change Management Framework can address organizational change challenges in your workplace?
S-14	How likely are you to recommend this exploration of the Change Management Framework to others?
S-15	What suggestions do you have for improving the experience of exploring the Change Management Framework?

**Data Collection and Analysis:** Using a predetermined evaluation checklist, this analysis concentrates on a selection of 96 research papers

**Fig. 2:**
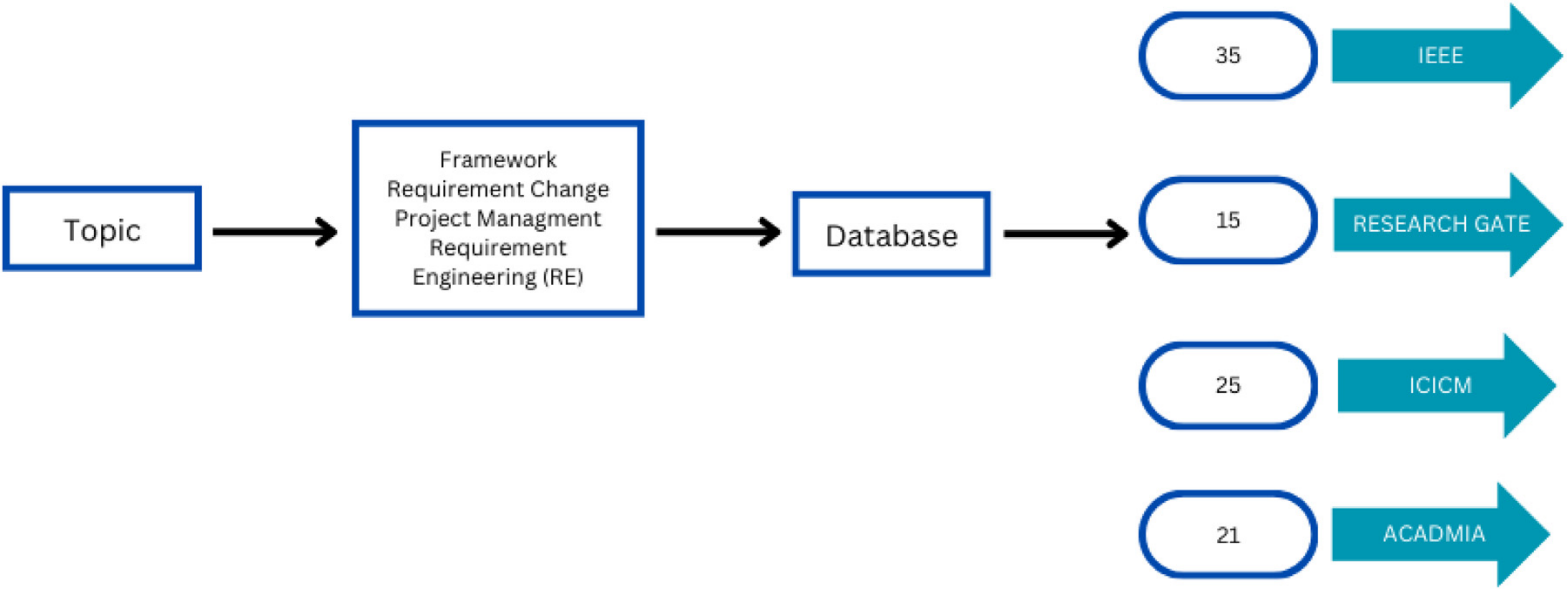
Challenges.

Fig. 2 List of different challenges identified from various publishers, including IEEE and ICICM, and identifies 5 papers that are pertinent for the survey given below.

The challenges identification analyses the different frameworks used in different research papers which are discussed through table and chart.

**Fig. 3:**
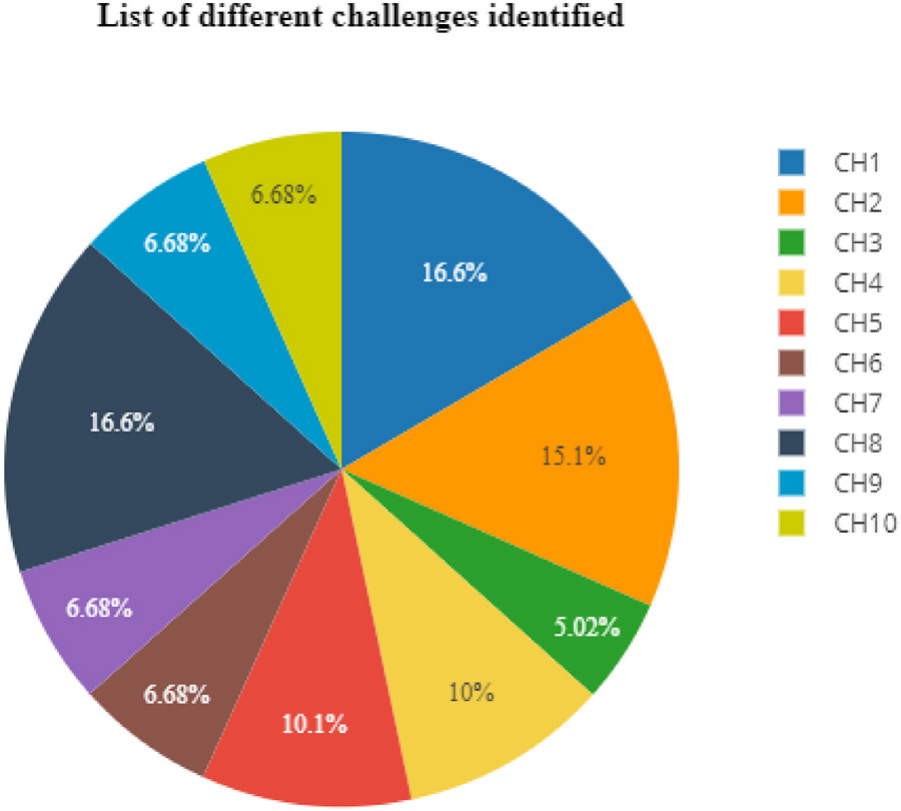

Fig. 3 List of different challenges identified the proposed review is focused upon the factors of change that are sensitive to the change. There are some steps as follows: Framework identifications compression concerning GSD, and Non-GSD frameworks identification of the affected requirements challenge.

**Table:** 

S No	Reference	Description	Framework
1	Business process change	(1). Imagine] (2). [update Participants, Reengineer groups] (3) [Identify] (4). [Restructure] (5). Rebuild (6). [System efficiency].	✓
2	Software requirements analysis and specification	(1). [Requirement] (2). Specification	✓
3	Review of job shop scheduling research and its new perspectives under Industry	(1).Initial training (2).Structural Environment(3).Workspaces (4). Categorization (5). Classification [idea improvement].	✓
4	Software project management with GAs	(1). Evaluate (2). Identify (3). Select (4). Design (5).Conduct (6). Develop	✓
5	An investigation of changing requirements with respect to development phases of a software project	(1). Unchanging requirements [stable requirements] (2). Modifying requirements [changeable, evolving requirements, resultant requirements, and flexible requirements].	✓
6	Requirement change management challenges in GSD	(1).Understand ability (2). Analyze (3). Changing (4).Implement.	✓
7	A Guide To The Project Management Body of Knowledge PMBOK Guide Sixth Edition	(1).Presentationattribute [Financial(Financialstability,Price,size),Service, Technical]	✓
8	A Software Engineering Framework for Achieving Long-Lasting Complex Systems	(1). ERP [to reduce negative result] (2). CSF of it to take the Edge off failing cases (3) racially balanced unified information system (4). SOS (5). Initiate (6). RE	✓
9	The change and evolution of requirements as a challenge to the practice of software engineering	(1). SDLC [contracted between user and developers]. (2) RE(3).Alternatives4 [initiatives](5). [life cycle factor, plan]	✓
10	The Role of Requirement Engineering in Software Development Life Cycle	(1)Changing requirements.(2).Phases.(3)Software project.(4) Statistical analysis	✓
11	A proposed framework for RC management in global software development [32]	(1). RCM (2). COMMUNICATION (3).LOCATION	✓
12	A Novel Framework for Change Requirement Management (CRM) In Agile Software Development (ASD)	(1). CRM, ASD (2). Initiate, (3). Evaluation (4) implement	✓
13	A Cloud-Based Framework For Managing Requirements Change In Global Software Development	(1). Single Platform (2).communicate collaborate (3). Control Managing (4) Individual.	✓
14	AZ-Model of software requirements change management in global software development	(1).RCM activities in Global Software Development (GSD) (2).AZ-Model of RCM”(2). limitations (3)Significant.	✓
15	Investigation of Project Administration related challenging factors of Requirements Change Management in global software development: A systematic literature review	(1)Challenges relates administration .(2) Geographical boundaries(3)failure ratio	✓

The proposed review is focused upon the factors of change that are sensitive to the change. There are some steps as follows: Framework identifications compression concerning GSD and Non-GSD frameworks identification of the affected requirements challenge.

**Traceability Analysis of Selection purpose and challenges:**

**Table:** 

R.P AP AR	CH1	CH2	CH3	CH4	CH5	CH6	CH7	CH8	CH9	CH10
**LR-1**	✓	✓		✓			✓	✓		
**LR-2**	✓	✓				✓		✓		✓
**LR-3**	✓					✓		✓		
**LR_4**	✓	✓		✓			✓	✓		
**LR-5**	✓			✓		✓	✓	✓		
**LR-6**	✓									
**LR-7**	✓			✓				✓		
**LR-8**										
**LR- 09**	✓	✓						✓		
**LR-10**	✓	✓			✓				✓	✓
**LR- 11**										
**LR- 12**	✓	✓	✓					✓		
**LR-13**	✓	✓	✓	✓				✓	✓	✓
**LR-14**		✓					✓	✓	✓	✓
**LR-15**		✓	✓			✓		✓	✓	

Fig. 3 presents an analysis of the framework's phases and the mean value of changing requirements, indicating that the Analysis Phase poses the highest challenge (66 %). The bar graph also shows a difference percentage of 34 %, while the lowest challenge is at 26.5 %. Table 1 provides a pictorial representation of the challenges faced in overall task performance.

**Table 1:** Challenges.

ID	Factor	Result
CH1	Change management	66 %
CH2	Project success and associate with the effectiveness	60 %
CH3	Estimate	20 %
CH4	Time	40 %
CH5	Geographically distributed	40.1
CH6	Requirement Uncertainty	26.6 %
CH7	Improving Business Process Reengineering (BPR) practices	26.6
CH8	Lack of communication	66 %
CH9	RCM in GSD	26.6 %
CH10	Culture difference	26.6 %

Modification between GSD and NON-GSD: This study focuses on requirement change management (RCM) as it examines the problems and potential solutions in global software development (GSD). It suggests models and frameworks to promote communication, enhance RCM operations, and deal with the complexity of GSD. In the context of GSD, the study emphasizes the significance of efficient teamwork, value-based valuation, and cloud-based platforms.

GSD AND NON-GSD IDENTIFICATION: The issues of change management in the Software Development Life Cycle (SDLC), notably with agile methodology and international teams, are highlighted in this literature study. It points out flaws in the current frameworks and suggests a thorough framework for successfully classifying, tracking, and managing requirements.

By employing this methodology, the study aims to gain insights into the effectiveness of the AHP approach, the management of change, the benefits and challenges of GSD, and the suitability of different development models for specific project requirements.

**Participant Demographics Table:** 

**Industry bar chart:** 

## Response summary

According to the survey results, most participants gave the Change Management Framework a positive impression, rating their comprehension and comfort level with its application as "Good" or "Excellent." Primarily garnering "Good" to "Excellent" evaluations, the training's efficacy, the content's relevancy, and the practical examples offered were highly valued. Nonetheless, a few categories, such participation and resource accessibility, shown variation, with some participants ranking these elements as "Average." This implies that although the training and the framework are useful, the integration of the framework into organizational procedures might be further enhanced by improvements in resource accessibility, engagement tactics, and support.

### Impact of CMF on data factory context

By standardizing and organizing data flows, the Common Model Framework (CMF) ensures uniform data transformation and integration procedures across multiple sources, which has an impact on the Data Factory environment. Data Factory pipelines can handle data with better quality, security, and compliance because to its assistance in defining and enforcing data governance principles. Data mapping, validation, and transformation rules are streamlined by CMF, which lowers complexity and improves scalability. As a result, data workflows are more effective, dependable, and maintainable, enabling businesses to use unified, correct data for analytics and decision-making procedures.

### Protocol validation

The research papers suggest future research directions in requirements change management, such as conducting more experimental studies, systematic literature reviews, and real-world case studies. These endeavors aim to deepen the understanding of requirement changes, develop practical frameworks, and evaluate the effectiveness of different approaches. Overall, the Papers contribute to the knowledge in requirements change management by offering insights into methodologies, tools, and techniques that can enhance project success rates, reduce costs, and improve the software engineering development process. While these approaches did not discourse much about the roles played by type and process in determining a successful change, they did draw special attention of change managers to the symbiotic relationship of human, technology and strategy in change process. According to the review, successful change is certainly a result of an effective strategy, which is reflected by the smooth way of various elements of change, at a given time.

The above-mentioned table has their own conclusions and future work which are summarized as follows.

The survey results in this study offer significant insights into the practical use and efficacy of the chosen change management framework, providing a data-driven view on its adoption and impact within businesses.

**Table:** 

S No	Survey Questions	Poor	Average	Good	Excellent
1	S-1	6.3 %	12.5 %	62.5 %	18.8 %
2	S-2	_	12.5 %	62.5 %	25 %
3	S-3	_	31.3 %	43.8 %	25 %
4	S-4	_	6.3 %	56.3 %	37.5 %
5	S-5	_	18.8 %	50 %	31.3 %
6	S-6	_	43.8 %	31.3 %	25 %
7	S-7	_	31.3 %	37.5 %	31.3 %
8	S-8	6.3 %	12.5 %	62.5 %	18.8 %
9	S-9	_	18.8 %	62.5 %	18.8 %
10	S-10	_	21.4 %	42.9 %	35.7 %
11	S-11	_	40 %	40 %	20 %
12	S-12	_	33.3 %	46.7 %	20 %
13	S-13	6.3 %	25 %	50 %	18.8 %
14	S-14	_	31.3 %	37.5 %	31.3 %
15	S-15	_	25 %	37.5 %	37.5 %

## Limitations

In the paper, numerous goals and purposes for study on software development, change management, and geographically dispersed systems are described. These goals are intended to address significant issues and advancements in the field of computer science. The study examines Business Process Reengineering (BPR) techniques and tools, recognizing their importance in completing software projects successfully.

It objectively assesses the success and failure rates of software projects, highlighting in particular how crucial it is to account for unforeseen changes throughout development. The importance of effective communication between users and designers is emphasized as a crucial goal, highlighting the need of effective communication for project success. The study emphasizes the importance of careful planning by attempting to create an accurate effect analysis to improve planning and the implementation of changes. In order to increase the effectiveness of the supply chain for software development, the research describes the qualities of Just-In-Time (JIT) vendors. The study uses a hospital as a case study to emphasize the interdependence of the design and testing phases and the need of good change management. With a focus on identifying communication risks and their effects, it suggests a methodology for Risk Communication Management (RCM) in geographically dispersed software development systems. The framework seeks to streamline the requirement management process by categorizing, tracking, saving, and updating requirements in a single repository. The main goal is to create a framework for effective RCM in a geographically dispersed software development environment, bringing stakeholders together on a single communication platform.

The “AZ-Model of RCM” presented in the study offers a thorough framework for GSD, aligning software development operations with global resources and cost-efficiency while highlighting the importance of efficient change management and communication.

By tackling the difficulties of change management, effective communication, and the complications of geographically distributed software development, the research seeks to improve software development processes. These goals highlight the value of effective project management and teamwork in the contemporary software development environment and jointly improve the field.

In conclusion, Global Software Development (GSD) has transformed to meet the demands of the modern IT industry, driven by the emergence of intelligent devices and applications. Agile methodologies have replaced outdated practices, enabling the dynamic nature of software development. However, managing requirement changes in distributed computing projects remains a significant challenge, compounded by communication barriers. Effective requirement management and stakeholder communication are vital for successful development. This study emphasizes the need for well-defined methodologies to navigate these challenges, recognizing the complexity of interactions in distributed environments. Addressing evolving requirements is crucial for project success. The comprehensive analysis highlights challenges in distributed software development and outlines strategies for managing changing requirements. In addition, the case study will be conducted to assess and improve the performance of the proposed model. All of them combined type, process and elements could shape a complete framework that lasts for contemporary and future change management practices.

The survey results show that participants have a moderate level of understanding and satisfaction with the Change Management Framework. While there is space for development in terms of comprehension and the overall efficiency of the training, the data also point to areas of strength. Participants are moderately confident in their ability to apply the framework to real-world circumstances, and many found the practical examples offered during training useful. This shows that the framework has the ability to effectively handle organizational transformation difficulties, while some tweaks may be required to improve its accessibility and involvement. Overall, these findings emphasize the significance of continuous refining and adaptation in change management training programs in order to better fulfil the requirements and expectations of participants.

## Discussion and conclusion

In the paper, numerous goals and purposes for study on software development, change management, and geographically dispersed systems are described. These goals are intended to address significant issues and advancements in the field of computer science. The study examines Business Process Reengineering (BPR) techniques and tools, recognizing their importance in completing software projects successfully.

It objectively assesses the success and failure rates of software projects, highlighting in particular how crucial it is to account for unforeseen changes throughout development. The importance of effective communication between users and designers is emphasized as a crucial goal, highlighting the need of effective communication for project success. The study emphasizes the importance of careful planning by attempting to create an accurate effect analysis to improve planning and the implementation of changes. In order to increase the effectiveness of the supply chain for software development, the research describes the qualities of Just-In-Time (JIT) vendors. The study uses a hospital as a case study to emphasize the interdependence of the design and testing phases and the need of good change management. With a focus on identifying communication risks and their effects, it suggests a methodology for Risk Communication Management (RCM) in geographically dispersed software development systems. The framework seeks to streamline the requirement management process by categorizing, tracking, saving, and updating requirements in a single repository. The main goal is to create a framework for effective RCM in a geographically dispersed software development environment, bringing stakeholders together on a single communication platform.

The “AZ-Model of RCM” presented in the study offers a thorough framework for GSD, aligning software development operations with global resources and cost-efficiency while highlighting the importance of efficient change management and communication.

By tackling the difficulties of change management, effective communication, and the complications of geographically distributed software development, the research seeks to improve software development processes. These goals highlight the value of effective project management and teamwork in the contemporary software development environment and jointly improve the field.

In conclusion, Global Software Development (GSD) has transformed to meet the demands of the modern IT industry, driven by the emergence of intelligent devices and applications. Agile methodologies have replaced outdated practices, enabling the dynamic nature of software development. However, managing requirement changes in distributed computing projects remains a significant challenge, compounded by communication barriers. Effective requirement management and stakeholder communication are vital for successful development. This study emphasizes the need for well-defined methodologies to navigate these challenges, recognizing the complexity of interactions in distributed environments. Addressing evolving requirements is crucial for project success. The comprehensive analysis highlights challenges in distributed software development and outlines strategies for managing changing requirements. In addition, the case study will be conducted to assess and improve the performance of the proposed model. All of them combined type, process and elements could shape a complete framework that lasts for contemporary and future change management practices.

The survey results show that participants have a moderate level of understanding and satisfaction with the Change Management Framework. While there is space for development in terms of comprehension and the overall efficiency of the training, the data also point to areas of strength. Participants are moderately confident in their ability to apply the framework to real-world circumstances, and many found the practical examples offered during training useful. This shows that the framework has the ability to effectively handle organizational transformation difficulties, while some tweaks may be required to improve its accessibility and involvement. Overall, these findings emphasize the significance of continuous refining and adaptation in change management training programs in order to better fulfil the requirements and expectations of participants

## Ethics statements

Our work did not involve data collected from social media platforms.

## Declaration of competing interest

The authors declare that they have no known competing financial interests or personal relationships that could have appeared to influence the work reported in this paper.

## Data Availability

Data will be made available on request.
